# The role of potassium ion channels in chronic sinusitis

**DOI:** 10.3389/fphar.2024.1431330

**Published:** 2024-07-01

**Authors:** Changhui Ding, Senxi Gai, Zhiyong Ma, Lizhuo Yang, Zhijie Fu

**Affiliations:** ^1^ Department of Otorhinolaryngology, The First Affiliated Hospital of Shandong First Medical University and Shandong Provincial Qianfoshan Hospital, Jinan, China; ^2^ The Key Laboratory of Cardiovascular Remodeling and Function Research, Chinese Ministry of Education, Chinese National Health Commission and Chinese Academy of Medical Sciences, Department of Cardiology, Qilu Hospital of Shandong University, Jinan, China

**Keywords:** chronic sinusitis, potassium ion channels, nasal mucosa, inflammatory reaction, nasal polyps

## Abstract

Chronic sinusitis is a common inflammatory disease of the nasal and sinus mucosa, leading to symptoms such as nasal congestion, runny nose, decreased sense of smell, and headache. It often recurs and seriously affects the quality of life of patients. However, its pathological and physiological mechanisms are not fully understood. In recent years, the role of potassium ion channels in the regulation of mucosal barrier function and inflammatory cell function has received increasing attention. In chronic sinusitis, there are often changes in the expression and function of potassium channels, leading to mucosal damage and a stronger inflammatory response. However, the related research is still in its early stages. This article will review the role of the potassium channel in the pathological and physiological changes of chronic sinusitis. The studies revealed that BK/TREK-1 potassium channel play a protective role in the nasal mucosal function through p38-MAPK pathway, and KCa3.1/Kv1.3 enhance the inflammatory response of Chronic rhinosinusitis by regulating immune cell function, intracellular Ca^2+^ signaling and ERK/MAPK/NF-κB pathway. Because ion channels are surface proteins of cell membranes, they are easier to intervene with drugs, and the results of these studies may provide new effective targets for the prevention and treatment of chronic sinusitis.

## 1 Introduction

Chronic rhinosinusitis (CRS) is a common chronic inflammatory disease that occurs in the nasal and paranasal sinus mucosa (Subspecialty Group of Rhinology, 2019; Fokkens et al., 2020). The overall incidence rate of Chinese people is approximately 8%, while that of European and American populations is about 10%–14% (Subspecialty Group of Rhinology, 2019; Fokkens et al., 2020). CRS often leads to nasal congestion, sticky or purulent nasal mucus with related imaging manifestations by MRI, CT and nasal endoscopy imaging manifestations (Subspecialty Group of Rhinology, 2019; Fokkens et al., 2020; Xie et al., 2024). Patients also suffer from reduced or lost smell and facial pain, which seriously affect the quality of life (Bai et al., 2024; Xie et al., 2024). Pharmacological treatment and surgical intervention are the primary strategies to alleviate the symptoms of CRS and preventing mucosal remodeling (Subspecialty Group of Rhinology, 2019; Fokkens et al., 2020; Bai et al., 2024; Xie et al., 2024). However, CRS cannot be fundamentally cured yet due to the complex pathogenesis.

The pathophysiological basis of CRS involves infiltration of inflammatory cells, aggregation of inflammatory cytokines, and remodeling of the nasal and sinus mucosa (Bai et al., 2024; Xie et al., 2024). Chronic inflammation related to CRS exhibits significant heterogeneity and is associated with multiple causes, including anatomical structures, environmental factors, and genetic elements (Bai et al., 2024; Sima et al., 2024; Xie et al., 2024). CRS is typically classified into two subtypes: CRS without nasal polyps (CRSsNP) and CRS with nasal polyps (CRSwNP) (Bai et al., 2024; Sima et al., 2024). CRSsNP is often accompanied by neutrophil infiltration and has a relatively good therapeutic effect, while CRSwNP is often accompanied by inflammation with high eosinophils and has a higher postoperative recurrence rate (Bai et al., 2024; Xie et al., 2024). However, the pathogenesis of CRS is not fully understood and there is a lack of targeted treatment. Therefore, in-depth research on the pathogenesis, prevention, and treatment of CRS is essential.

In recent years, the role of ion channels in regulating the mucosal barrier and inflammatory cell function has been increasingly recognized (Pelletier and Savignac, 2018; Backaert et al., 2021; Deng et al., 2021b). As the most widely distributed ion channels in the human body, potassium ion channels play a crucial role in the formation of excitable cell action potentials, neurotransmitter transmission, and electrical activity in the myocardium and skeletal muscles (Sesti et al., 2023; Xia et al., 2023). In nonexcitable cells, increasing research has found that potassium ion channels can regulate cell volume, stretch, and intracellular calcium, and participate in proliferation, immune response, and hormone secretion, etc (Sesti et al., 2023; Xia et al., 2023). However, there are only a few initial studies on the relationship between potassium channels and CRS. In the following review, we summarize the physiological and pathological contributions of potassium channels to CRS based on the current literature.

## 2 Molecular composition, characteristics, and physiological actions of potassium ion channels

**FIGURE 1:**
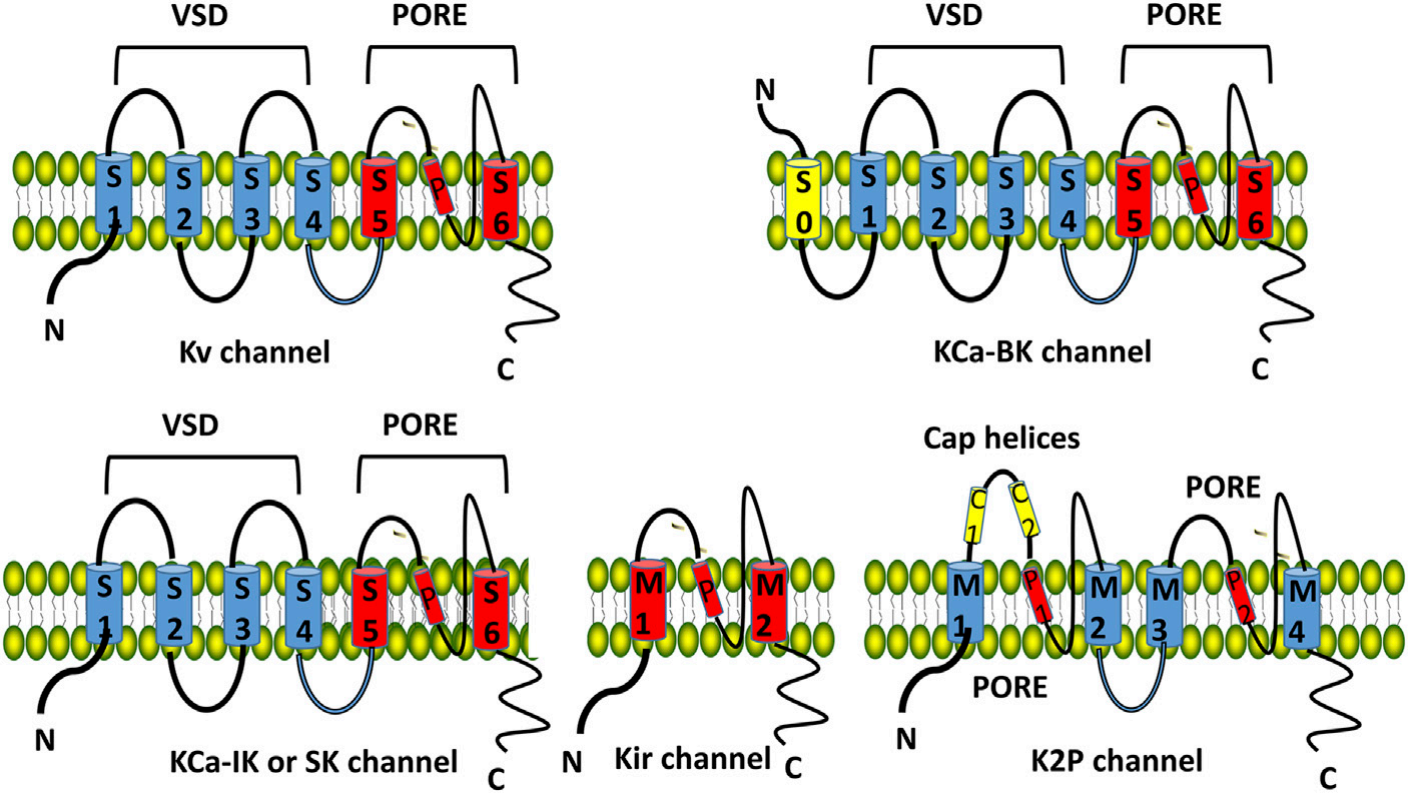
Structure and classification of potassium channels. The Kv, KCa-IK and KCa-SK channel consists of six transmembrane domains (TMDs, S1–S6 from the N-terminus) and 1 spiral pore domain (located between S5 and S6). The first four TMDs (S1–S4) form the voltage sensing domain (VSD). The last two TMDs (S5–S6) form the pore domain. KCa-BK consists of 7 TMDs and 1 spiral pore. Kir consists of 2 TMDs and 1 spiral pore. K2P K^+^ channel has 4 TMDs (M1-M4), 2 spiral pore domains (P1, P2), and a specific extracellular cap helix domain (cap helices C1, C2). Kv, voltage gated K^+^ channels; Ki, inwardly rectified K^+^ channels; KCa, calcium-activated potassium channels; K2P, two pore domain K^+^ channels.

Human genome sequencing has identified more than 80 potassium channel genes, which are mainly divided into four subgroups based on transmembrane topology and activated modes: voltage gated K^+^ channels (Kv), inwardly rectified K^+^ channels (Ki), two pore domain K^+^ channels (K2P), and calcium-activated potassium channels (KCa) (Figure 1) (Sesti et al., 2023; Xia et al., 2023).

### 2.1 Kv

Kv consists of 6 transmembrane domains (TMDs, S1-S6 from the N-terminus) and 1 spiral pore domain (located between S5 and S6), the first 4 TMDs (S1-S4) form the voltage sensing domain (VSD, Figure 1). Kv family includes 12 channels: Kv 10 is the ether go (EAG) K^+^ channel, Kv 11 is the human ether a go related gene (hERG) K^+^ channel, and Kv 12 belongs to the EAG family (Sesti et al., 2023; Xia et al., 2023). Kv 5, Kv 6, Kv 8, and Kv 9 are members of the electrically silent Kv subfamilies which must form functional heterotetramers with Kv 2 (Kv2/KvS) (Bocksteins, 2016)11. Kv is involved in the generation of action potentials and synaptic transmission and maintains the normal electrophysiology of the heart and the nervous system (Bocksteins, 2016; Sesti et al., 2023; Xia et al., 2023).

### 2.2 KCa

KCa are ligand-gated K^+^ channels and classified into big conductance KCa (KCa 1.1/BK), intermediate conductance KCa (KCa 3.1/IK), and small conductance KCa (SK) based on their single channel conductivity characteristics (Sancho and Kyle, 2021; Xia et al., 2023)^[^. BK consists of 7 TMDs and 1 spiral pore (Figure 1), which are mainly distributed in the central nervous system and maintain the electrophysiological balance. The structure of IK and SK is similar to that of Kv (Figure 1). IK primarily affects blood and lymphatic tissues, regulating procoagulant activity and platelet cell volume of platelets; SK is involved in synaptic plasticity, cognitive function, and cardiac repolarization processes (Sancho and Kyle, 2021; Forzisi and Sesti, 2022; Xia et al., 2023).

### 2.3 Kir

Kir consists of 2 TMDs and 1 spiral pore (Figure 1), including classic Kir 2, 3, 6 channels, and potassium transport channels (Kir 1, 4, 5, 7) 11. Kir 3 is associated with G protein activation, and Kir 6 is an ATP sensitive potassium channel. Kir is involved in the formation of action potentials in the myocardium and skeletal muscles (Forzisi and Sesti, 2022; Xia et al., 2023).

### 2.4 K2P

It has 4 TMDs (M1-M4), 2 spiral pore domains (P1, P2), and a specific extracellular cap helix domain (cap helices C1, C2, Figure 1), including 6 subtypes: TWIK, TREK, TASK, THIK, TALK and TRESK (Forzisi and Sesti, 2022; Xia et al., 2023). K2P1.1 (TWIK-1), K2P3.1 (TASK-1), K2P9.1 (TASK-3), and TASK-1 are expressed in the cardiovascular system and are involved in regulating cardiac rhythm and myocardial cell function; K2P 2.1 (TREK-1) is expressed primarily in the nervous system, involved in neuronal excitability, synaptic plasticity, and cognitive function (Forzisi and Sesti, 2022; Xia et al., 2023).

The primary function of potassium channels is to mediate the transmembrane movement of potassium ions, regulate cell polarization, and maintain the resting potential and action potential formation in excitable cells (Forzisi and Sesti, 2022; Xia et al., 2023). An increasing number of studies have also unveiled the nonconducting functions of potassium channels (Forzisi and Sesti, 2022). On the one hand, they can modulate intracellular calcium concentration by influencing the membrane potential of nonexcitable cells leading to changes of calcium-dependent signaling pathways; on the other hand, they can directly activate signaling pathway proteins to regulate fundamental cell functions, such as proliferation, migration, apoptosis, and cytokine secretion (Forzisi and Sesti, 2022; Gong et al., 2023).

## 3 The role of potassium ion channels in mucosal remodeling of chronic sinusitis

Nasal and sinus mucosal remodeling is an important pathophysiological basis for CRS, including epithelial cell damage, squamous metaplasia, and an increase in microvillus and goblet cells (Gong et al., 2023; Xie et al., 2024), but the molecular mechanisms are not fully understood.

Previous studies have found that BK potassium channels are the main surface-expressing potassium channels in the airway epithelium, including the nasal and sinus mucosa (Manzanares et al., 2011; Bengtson et al., 2021). qPCR and patch clamp currents confirmed that BK channels were abundantly expressed in apical membrane of ciliated cells rather than basal cells and goblet cells (Manzanares et al., 2011; Bengtson et al., 2021). Long-term inhibition of BK channels or knockdown of the BK a-subunit KCNMA1, lead to airway surface dehydration, which suggested the critical role of BK in maintaining airway surface liquid (ASL). Furthermore, inflammatory cytokines IFN-γ and TGF-β1 could decrease expression and activity of BK channel in epithelial cells and caused ASL depletion, which could be reversed by the BK opener or overexpression of BK (Manzanares et al., 2014). Nasal samples expressed γ subunit leucine-rich repeat-containing protein 26 (LRRC26) of BK channel, participating in Ca^2+^-activated Cl^−^ secretion in nasal epithelia (Bengtson et al., 2021). The possible mechanism may be that functional BK channels at the apical membrane of epithelial cells create the electrochemical gradient necessary for Cl^−^ secretion through Ca^2+^-activated Cl^−^ channel. *In vitro* experiments, cigarette smoke, an important pathogenic factor for CRS, can cause a significant increase in mRNA expression of inflammatory factor *TGF-β1* and MMP-9 activity and decrease of CFTR and BK channel activities in primary human airway epithelial cells, thus reducing ASL volumes and increasing mucus concentrations (Sailland et al., 2017; Kim et al., 2021). Inhibition of signaling pathways reveals that BK activity is decreased via the p38 cascade. In addition, pre-treatment with pirfenidone, a drug presently used to inhibit TGF-β signaling, ameliorated BK dysfunction and ASL volume loss (Sailland et al., 2017). Therefore, BK potassium channels may be inhibited or donregulated by CRS pathogenic factors leading to mucociliary dysfunctionand be regulated by p38-MAPK/TGF-β pathway.

K2P potassium channels can be activated or blocked by a variety of physiological stimuli, including mechanical stimuli, taste, temperature, and pH, which are factors affecting the progression of CRS (Graudenz et al., 2006; Sesti et al., 2023; Xia et al., 2023). K2P potassium channels including KCNK1 (TWIK-1), KCNK2 (TREK-1), KCNK5 (TASK-2) and KCNK6 (TWIK-2) were expressed in respiratory epithelial cells and modulated Na^+^ absorption and Cl secretion in epithelial cells, which are the main components of ASL (Zhao et al., 2012; Schwingshackl et al., 2013). The bitter taste receptor has been shown to play a role in the pathogenesis of CRS by enhancing upper airway innate immunity (Takemoto et al., 2023). In polarized sinonasal epithelial cells of CRS patients, the bitter taste receptor agonist, denatonium, decreases the epithelial K2P current through a cAMP-dependent signaling pathway (Kohanski et al., 2021). In a clinical study, TREK-1 is expressed in the normal sinus mucosa and is located in the superficial epithelial layer, the submucosal glands, and the endothelial cells. TREK-1 expression decreased significantly in both CRSsNP and CRSwNP, without significant differences between the 2 disease groups (Kim et al., 2018). Knockdown of TREK-1 using specific siRNA or blocking of TREK-1 with fluoxetine can increase the permeability of epithelial and endothelial cells (Kim et al., 2018).

In a closely related disease, allergic rhinitis, TREK-1 expression levels were significantly decreased in patients with allergic rhinitis and rats with ovalbumin-induced nasal allergy than their normal controls (Jiang et al., 2015). CRS pathogenic cytokine, interleukin (IL)-4, suppressed the expression of Trek1 in the nasal mucosa via up regulating the expression of the histone deacetylase (HDAC)1. Inhibiting HDAC1 improve nasal epithelial barrier dysfunction (Jiang et al., 2015). Furthermore, knockdown of Trek1 could lead to intestinal epithelial barrier disruption (Huang et al., 2016). Proinflammatory cytokines including IL-4, IL-5, IL-13, or TNF-α suppressed the expression of Trek1, activated p38 MAPK pathway and HDAC1 (Jiang et al., 2015; Huang et al., 2016). The inhibitors of p38 or HDAC1 abolished proinflammatory cytokine induced suppression of Trek1. In clinical therapy, antigen-specific immunotherapy (SIT) could restore the expression of TREK-1 in the nasal epithelia that was suppressed in patients with nasal allergy (Wang et al., 2015). These results suggested that TREK-1 channel may be inhibited by pathogenic cytokine in CRS resulting in epithelial barrier disruption and be regulated by p38-MAPK/HDAC1 pathway.

## 4 The role of potassium channels in the inflammatory response of CRS

**FIGURE 2:**
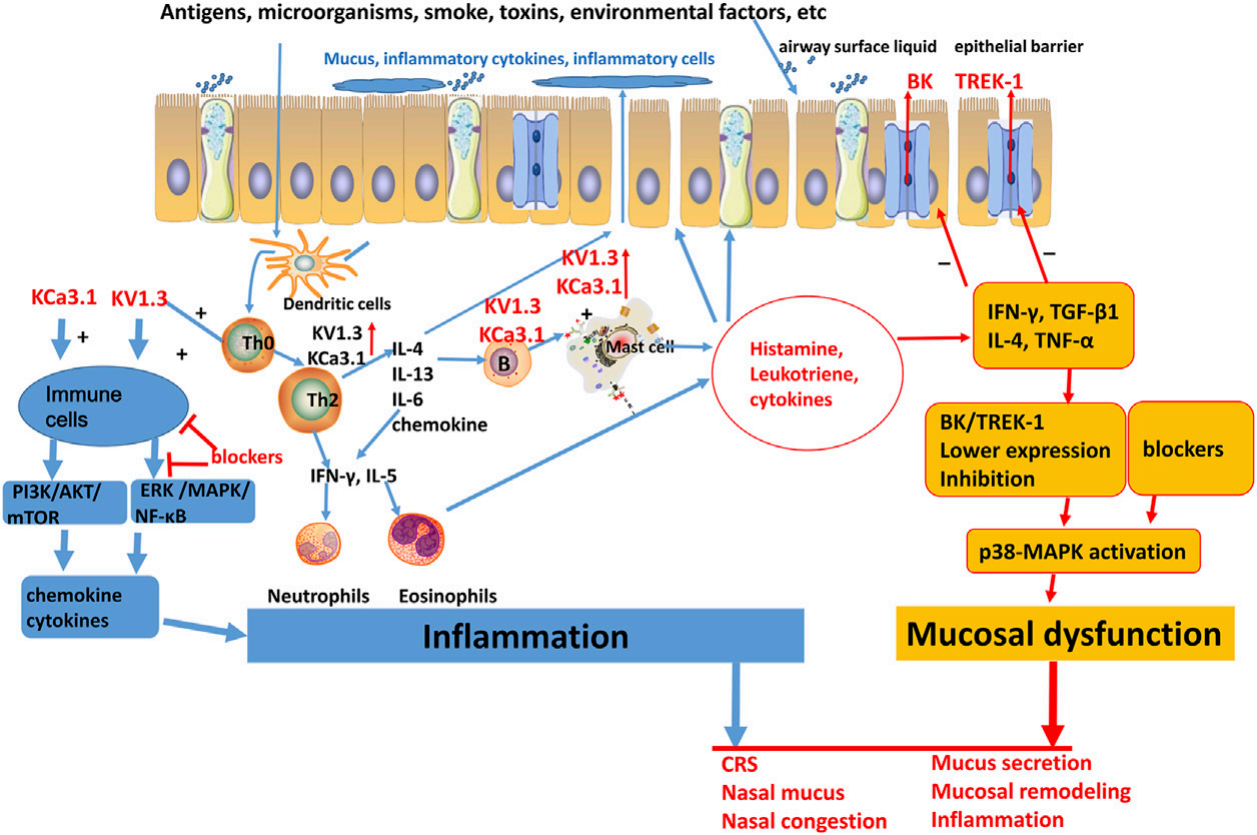
Schematic plot of the role of potassium channels in chronic sinusitis. BK, big conductance calcium-activated potassium channels; TREK-1, TWIK—related K^+^ channel 1; KV, voltage gated K^+^ channels; KCa, calcium-activated potassium channels; IL, interleukin; IFN-γ, interferon-γ; TGF-β, transforming growth factor-β; TNF-α, tumor necrosis factor-α.

Inflammation is a crucial driver of the onset and progression of CRS, with multiple inflammatory cells and cytokines that amplify the intensity of the inflammatory response in CRS (Subspecialty Group of Rhinology, 2019; Fokkens et al., 2020; Bai et al., 2024; Xie et al., 2024). However, the precise regulatory mechanisms remain largely unclear. Increasing evidence indicates that ion channels play a significant role in immune cell function and inflammatory response (Pelletier and Savignac, 2018; Backaert et al., 2021; Deng et al., 2021b). Especially potassium ion channels are widely distributed and expressed in various immune cells and are involved in antigen presentation, cell migration, phenotype transformation, and the production of inflammatory cytokines (Pelletier and Savignac, 2018; Sesti et al., 2023; Xia et al., 2023). Previous studies have revealed that KCa3.1 and KV1.3 are present in T lymphocytes, and their relative expression depends on the type of T cells, including naive memory, effector memory, and central memory (Chen et al., 2024; Sima et al., 2024). The potassium channel ERG1 and KV1.3 are involved in the maturation and differentiation of B lymphocytes (Sima et al., 2024). KV1.3, KV1.5, KCa3.1, Kir2.1/2.2, Kir6.1/6.2 and TWIK2, TREK1 can be expressed in monocytes and macrophages, regulating phagocytic function and secreting inflammatory cytokines (Man et al., 2023). Mast cells and eosinophils can also express potassium channels KCa3.1 and Kv potassium channels (Lin et al., 2015). These studies indicate that KCa3.1 and KV1.3 are the channels of potassium ions expressed abundantly in immune cells, and their opening leads to hyperpolarization of the cell membrane, restricting the flow of calcium ions, thus modulating intracellular signaling pathways and regulating immune cell function (Figure 2).

In the inflammatory response of CRS, related inflammatory cytokines can reduce the expression of the TREK-1 potassium channel, while blocking the TREK-1 channel can further promote inflammatory cytokine-induced nasal mucosal epithelial permeability and lymphocyte trans-endothelial infiltration (Kim et al., 2018). Therefore, TREK-1 provides a protective effect on inflammatory response of CRS.

The role of other potassium ion channels in the inflammatory response of CRS has not yet been reported. However, CRS is closely related to allergic rhinitis and asthma, with 25%–70% of patients with CRS having concomitant allergic rhinitis, especially patients with CRSwNP and CRS with increased eosinophils that have a higher tendency to concomitant allergic rhinitis. 21%–47% of CRSsNP patients may have concurrent asthma symptoms, while CRSWNP with increased eosinophils may have a prevalence of up to 60% of concurrent asthma (Grimm et al., 2023). Therefore, CRS, allergic rhinitis, and asthma share certain common mechanisms of inflammatory response, and the role of potassium ion channels in the inflammatory response of allergic rhinitis and asthma can provide clues for CRS.

In mice with allergic rhinitis, Lin et al. found that lentiviral KCa3.1-shRNA could decrease the number of goblet cells and mast cells in nasal mucosa and the levels of the inflammatory cytokines IL-4, IL-9, and IL-17 in nasal lavage fluid. In mast cells lentiviral KCa3.1-shRNA decreased KCa3.1 expression and inhibited the secretion of IL-6, IL-8 and PI3K/AKT activity, which was enhanced by the PI3K pathway inhibitor LY294002 (Lin et al., 2015). The absence of KCa3.1 in T lymphocytes, B lymphocytes, mast cells, and bronchial smooth muscle cells provides a protective effect for OVA induced asthma (Kazama et al., 2015; Pelletier and Savignac, 2018). In the immune cells, pharmacological inhibition of KCa 3.1 decreased the expression of IFN-γ in CD4^+^ T cells, chemokine-induced dendritic cell migration and monocyte chemotaxis by the downregulation of migration markers (CCR5 and CCR7), reduced the expression and secretion of pro-inflammatory cytokines IL-6 and IL-8 in mast cells, which might be regulated by the PI3K/AKT/mTOR signaling pathway (Ohya and Kito, 2018). These results suggest that inhibition of KCa3.1 may reduce the inflammatory response of allergic disease via PI3K/AKT/mTOR signaling pathway.

In asthmatic mice, the expression of Kv1.3 in lung tissues and the current intensity of Kv in CD4^+^ T cells was significantly higher than the normal control (Zhou et al., 2018). The selective Kv1.3 blocker PAP-1 reduces airway hyperresponsiveness, inflammatory cell counts in bronchoalveolar lavage fluid and serum, and alleviates airway inflammation (reducing IL-4 and IL-17 levels) by inhibiting the ERK-NF-κB signaling pathway (Zhou et al., 2018). In a rat model of asthma, ShK-186, a selective KV1.3 channel blocker, reduced levels of IL-4 and IL-5 in bronchoalveolar lavage fluids and inhibited the proliferation of ovalbumin-specific T cells (Koshy et al., 2014). In ox-LDL-treated macrophages, Kv1.3-siRNA attenuated the expression of IL-6 and TNF-α and reduced the phosphorylation of ERK1/2 and NF-κB in macrophages (Zhang et al., 2022). However, Kv1.3-siRNA could not inhibit inflammation during treatment with PD-98059, a specific inhibitor of ERK (Zhang et al., 2022). KV1.3 blocker PAP-1 impaired intracellular Ca2+ signaling of neutrophils and their recruitment during inflammation (Immler et al., 2022). In Jurkat T cells, a selective blocker, FS48 significantly suppressed Kv currents, Ca^2+^ influx, MAPK/NF-κB/NFATc1 pathway activation, and TNF-α and IL-2 production (Deng et al., 2021a). Therefore, inhibition of Kv1.3 may reduce the inflammatory response by regulating immune cell function, intracellular Ca^2+^ signaling and ERK/MAPK/NF-κB pathway.

In summary, TREK-1 channel may be suppressed and KCa3.1, KV1.3 may be upregulated in CRS resulting in immune cell activation and inflammatory response. Intracellular Ca^2+^ signaling and ERK/MAPK/NF-κB pathway may act as a downstream regulator of potassium channels in CRS. (Figure 2).

## 5 Potential targeting potassium channels for CRS

Although increasing evidence suggests that potassium channels may be promising molecular targets for treating CRS, one important hindrance is that highly specific blockers for potassium channels are unavailable. *In vitro* experiments, BK channel blocker paxilline, TREK-1 blocker fluoxetine and knockdown of BK and TREK-1 can lead to nasal mucosal dysfunction, which suggests the specific opener to be a potential target for overcoming mucosal remodeling of CRS (Manzanares et al., 2011; Manzanares et al., 2014; Kim et al., 2018). Gene silence of KCa 3.1 and Kv1.3 may reduce the inflammatory response and immune cell function in animal and cellular experiments. Especially, selective Kv1.3 blockers PAP-1 and ShK-186 showed significant protective effect on OVA induced airway hyperresponsiveness and inflammation (Koshy et al., 2014; Lin et al., 2015; Ohya and Kito, 2018; Zhou et al., 2018). However, these potassium channel blockers and openers has not yet been explored in patients with CRS. The main reseaon is that high affinity inhibitors or openers are either small organic molecules or peptides isolated from venomous animals resulting in severe adverse or unsafe drug effect (Varga et al., 2021). Interestingly, fluoxetine a clinically prescribed antidepressant can inhibit TREK-1 (Kim et al., 2018), losartan reduced cigarette smoke-induced airway inflammation via inhibiting BK channel (Kim et al., 2021), lovastatin blocked Kv1.3 channel in human T cells (Zhao et al., 2015), which point out the commonly used drugs acting as prospective regulators for potassium channels in CRS. A Placebo-Controlled Trial published in 2024 report that a novel TASK channel antagonist nasal spray improve upper airway collapsibility in patients with obstructive sleep apnea (Osman et al., 2024). The result suggests that nasal spray consisting of potassium channel blocker or opener may be a more practical new therapy to avoid their adverse effect just like routine use of corticosteroid nasal spray in CRS.

## 6 Conclusion

Chronic sinusitis is one of the most common chronic diseases and greatly impairs the quality of life of millions of patients worldwide. Pharmacological and surgical treatments can alleviate symptoms, but the patients often suffer from the reoccurrence and repeated treatment. Therecent studies revealed that BK/TREK-1 potassium channel play a protective role in the nasal mucosal function through p38-MAPK pathway, and KCa3.1/Kv1.3 enhance the inflammatory response of CRS by regulating immune cell function, intracellular Ca^2+^ signaling and ERK/MAPK/NF-κB pathway. These results suggest that the selective openers or blockers for the potassium channels may provide a promising new target for the prevention and treatment of CRS. However, these studies mainly focus on *in vitro* experiments and there is a lack of effective animal models for CRS. Additional research should be conducted to understand the intricate mechanisms underlying the role of potassium channels in CRS from clinical studies, and design new regulators to be used in clinical practice with less adverse effect. It is a convenient to find whether the frequently prescribed drugs have effect on potassium channels and to design nasal spray for the channel regulators.
